# State Registration in America

**Published:** 1916-05-06

**Authors:** 


### STATE REGISTRATION IN AMERICA.

It is not generally known that the idea of State Registration originally reached the United States from England. We are indebted to Dr. Hurd, of Baltimore, the eminent authority, for the following interesting facts: State Registration was first suggested in the United States by Sir Henry Acland on his visit in 1874, but the movement did not have special force in America until the year 1903, at which time the States of North California, New Jersey, Virginia, and New York passed more or less satisfactory laws in reference to the registration of nurses. The State of Maryland had its law enacted in 1904.

There were thirty-nine States in 1914 in which there were laws relating to the practice of nursing. In eight of these registration is compulsory—that is, no woman can practise as a registered nurse unless she has submitted to an examination by a State Board. In thirty-one States the law is permissive rather than compulsory. In these States it is possible for a woman to practise as a nurse—even as a trained nurse or a qualified or certified nurse, provided that she does not use the words "Registered Nurse," or the abbreviation "R.N." after her name. This permits of the existence of correspondence schools, commercial hospital schools, and short-term schools.

In Maryland registration is not compulsory, and most of the smaller schools in special hospitals have not yet sent up their graduate nurses for examination and registration. More nurses, however, are availing themselves of registration each year.

In States where even the permissive law is the best which can be obtained, the effect upon the education of nurses, the course of study in the training schools, and the attainment of a higher standard of nursing care for the sick has been excellent. In New York especially, where no examination can be taken by the graduates of any school out of the State except the school be registered and approved by the Board of Regents at Albany, New York, such registration has proved of the greatest service in improving schools generally throughout the country. And such also has been the result of registration to a less degree in other States. In New York, to obtain registration a school must have certain definite preliminary educational requirements, a minimum course of study, and must submit to inspection. Affiliation of other schools from New York has also been promoted by the desire of their students to fit themselves to pass the New York examination. In Bellevue Hospital alone, over thirty schools from New York and other States are represented by students who are pursuing courses of greater or less duration to fit themselves for registration.

The effect throughout the country has been to produce more experienced nurses, shorter hours, better living conditions, better food, more uniform entrance requirements, and to place the instruction of nurses on an educational basis. The Red Cross (Rural) nurses, Army nurses, and School nurses are all chosen in New York from registered nurses.

